# Excited-state chemistry of the nitromethane anion mediated by the dipole-bound states revealed by photofragment action spectroscopy†

**DOI:** 10.1039/d3sc04342h

**Published:** 2023-10-16

**Authors:** Sejun An, Dabin Kim, Junggil Kim, Sang Kyu Kim

**Affiliations:** a Department of Chemistry, KAIST Daejeon 34141 Republic of Korea sangkyukim@kaist.ac.kr

## Abstract

We report the first experimental observation of the excited dipole-bound state (DBS) of the cryogenically cooled nitromethane anion (CH_3_NO_2_^−^), where the excess electron is loosely attached to the singlet or triplet neutral-core. Photofragment and photodetachment action spectra have been employed for the dynamic exploration of Feshbach resonances located even far above the electron detachment threshold, giving excitation profiles from the ground anionic state (D_0_) to the DBSs which match quite well with the spectral structures of the photoelectron spectra. This indicates that the electron transfer from the nonvalence orbital (of DBS) to the valence orbital (of anion) is mainly responsible for the anionic fragmentation channels, giving strong evidence for that the DBS plays a dynamic doorway-role in the anionic fragmentation reactions. Photofragment action spectra have also been obtained for the anionic clusters of (CH_3_NO_2_)_2_^−^, (CH_3_NO_2_)_3_^−^, or (CH_3_NO_2_·H_2_O)^−^, giving the relative yields of various fragments as a function of the excitation energy for each cluster. The absorption profiles of the anionic clusters exhibit substantial blue-shifts compared to the bare nitromethane anion as their ground states are much stabilized by solvation. The anionic fragmentation pattern varies among different clusters, giving essential clues for the thorough understanding of the whole anionic dynamics such as the dynamic role of the short-lived nonvalence-bound states of the clusters.

## Abstract 2

Dipole-bound states of the cryogenically cooled nitromethane anion which are associated with the S_0_, T_1_ or T_2_ state of the neutral-core give rise to anionic fragments.

## Graphical abstract

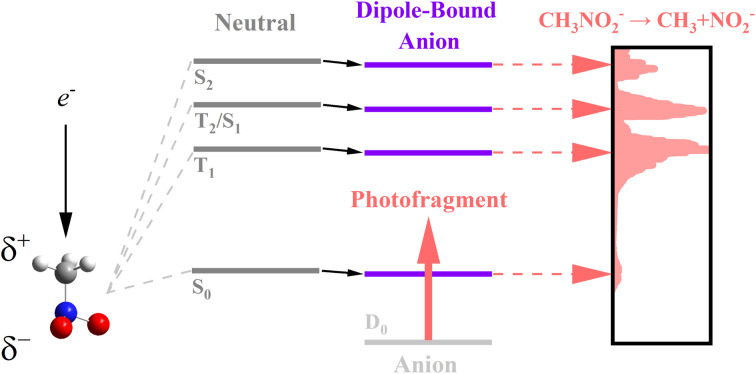

## Introduction

Entry or exit of the electron in the respective form of electron-attachment or electron-detachment is the fundamental step in the anion chemistry or physics. Actually, movement of the electron in and out of the anionic system is ubiquitous and essential in a number of chemical^1^ and biological^2^ functional processes of redox reactions, dissociative electron attachment (DEA),^3^ or formation of interstellar species.^4,5^ It is quite common that the excess electron in the stable anion occupies one of the vacant valence orbitals of the otherwise neutral molecule, giving rise to significant changes of the geometrical structure and chemical reactivity whereas the electron affinity estimated from the photoelectron spectroscopy of the anion represents how strongly the electron is bound to the neutral core. On the other hand, since its first conception by Fermi and Teller,^6^ the nonvalence-bound state (NBS) where the excess electron is only loosely bound to the neutral-core without occupying the valence orbital has long been intensively investigated for many recent decades. NBSs, according to the nature of the electron binding potential, may be classified into the dipole-bound state (DBS),^7,8^ quadruple-bound state (QBS),^9^ or correlation-bound state (CBS).^10^ Although the electrostatic potential such as charge–dipole or charge–quadruple interaction is considered to be mainly responsible for the electron binding of the DBS or QBS, respectively, the quantum-mechanical correlation effect and/or the charge-induced-dipole interaction (due to the large polarizability) could also contribute quite significantly in a cooperative way. Specifically, recent dynamic^11–15^ and spectroscopic^9,16–21^ studies of NBSs under cryogenically cold conditions^22^ have triggered the molecular-level investigation of the electron-binding nature of NBSs.

While the recently developed time-resolved electron-detachment dynamic studies^10–15,23–25^ (*e.g.* autodetachment) at well-defined Feshbach NBS resonances turn out to be extremely useful for disentangling the electron-binding dynamics, the experimental approach to unravel the dynamic role of the NBS as the doorway to various anionic (half- or full-collisional) reactions seems to be still in infancy. In this regard, a very recent study^13^ on the anionic fragmentation reaction mediated by the DBS of *ortho*-, *meta*- or *para*-iodophenoxide is quite noteworthy. Therein, the I^−^ fragment action spectra reflect the quantum resonance structures of DBSs whereas the state-specific fragment yields give the efficiency of the anionic fragmentation channel compared to that of the competitive autodetachment process. This may belong to a new form of DEA as the excess electron loosely attached to the positive end of the dipole (of the DBS prepared by the optical excitation) may be transferred to the specific valence orbital leading to either the prompt rupture of the particular chemical bond or rather slow anionic chemical-bond dissociation taking place on the vibrationally hot anionic state. It is interesting to note that the anionic fragmentation pathway mediated by the DBS (prepared by the optical excitation) could be finely controlled in terms of the energy given to the system as well as associated quantum-mechanical states in contrast to the more traditional electron-collision experiment of DEA. In this regard, photofragment excitation (PHOFEX) spectroscopy where the specific anionic fragment yield is monitored as a function of the excitation energy could be an extremely useful tool for unravelling the nature of the dynamic doorway states to the anionic fragmentation channels, as successfully demonstrated in the recent report on the reaction dynamics of the *ortho*-, *meta*- or *para*-iodophenoxide anions (*vide supra*). PHOFEX spectroscopy has great advantages over photodetachment spectroscopy (where the photoelectron yield is monitored as a function of the excitation energy) in terms of its little background signal as the directly ejected photoelectron in the latter persists and even increases with increasing the excitation energy above the electron-affinity threshold whereas the absorption profile of the anion species is supposed to be straightforwardly reflected in PHOFEX spectroscopy. Furthermore, it would be even ideal for the investigation of the excited-state chemistry over the wide excitation energy region even far above the detachment threshold,^26–29^ if and only if it is associated with any fragmentation channel in the overall anionic reaction. Despite these advantages (compared to other conventional photoelectron and photodetachment spectroscopic methods), PHOFEX spectroscopy has been surprisingly rarely employed for the chemical dynamics study of the anion, mainly because of the extremely low yields of the anionic fragments at the excitation energy above the photodetachment threshold.

Herein, we have investigated the excited-state chemistry of the nitromethane anion (CH_3_NO_2_^−^) using PHOFEX spectroscopy in addition to the photoelectron and photodetachment spectroscopic methods. The nitromethane anion is one of the most-studied radical anion systems^30–36^ whereas the presence of its DBS, as the dipole moment of nitromethane is 3.46 Debye,^30^ has been demonstrated on several occasions beforehand. Probably due to the very short lifetime, however, Feshbach resonances of the DBS of the nitromethane anion have not yet been observed. As a matter of fact, from the (I·CH_3_NO_2_)^−^ cluster study, the Neumark group^25,37^ reported that the nitromethane DBS anion survives only briefly (*τ* ∼400 fs) before it relaxes into the valence anionic states. The direct excitation spectrum to the DBS from the anionic ground state thus has not been reported to date, hampering the further reaction dynamics study of the current system. In this work, by employing PHOFEX spectroscopy, we were able to unravel the absorption profiles reaching the DBSs associated with the ground (S_0_) and several excited (T_1_, S_1_, T_2_) states of the neutral-core over the wide excitation energy range as well as the state-specific fragmentation patterns for the first time. PHOFEX spectroscopic study for the anionic clusters of (CH_3_NO_2_)_2_^−^, (CH_3_NO_2_)_3_^−^, or (CH_3_NO_2_·H_2_O)^−^ gives unprecedented information regarding the energy flow leading to the solvent evaporation and/or chemical-bond dissociation, which is most likely mediated by the electron conveyance among nonvalence and valence orbitals of the anion. Anion PHOFEX spectroscopy turns out to be a truly excellent experimental tool for unravelling the anionic chemical dynamics especially regarding anionic fragmentation reactions resulting from the electron transfer among nonvalence and valence molecular orbitals of the anionic systems.

## Experimental and computational methods

### Experimental methods

To generate nitromethane cluster anions, neon gas was mixed with liquid nitromethane and its clusters, and then expanded through a pulsed Even-Lavie valve coupled to a filament ionizer. Secondary electrons were produced by the electron ionization of the neon molecular beam and were attached to the nitromethane and its clusters. The generated anions passed through a skimmer into a quadrupole deflector that guides the anions orthogonally and subsequently through an RF quadrupole mass filter (Ardara Technologies) for initial mass selection. The mass-selected ions entered a quadrupole ion trap held at 25 K, where they were stored for 10–49 ms and cooled *via* collisions with He buffer gas (99.999%). The internally cooled anions were accelerated into the time-of-flight region, and then the mass selected anions were intersected by nanosecond and femtosecond laser pulses in the velocity-map photoelectron imaging spectrometer. Photoproducts (electrons or fragments) generated by the laser pulses were detected by chevron-type microchannel plates (MCPs) backed by a phosphor screen. The photoelectron images were recorded by a CMOS camera, while the photodetachment and PHOFEX spectra were recorded by a photomultiplier tube (PMT). Photoelectron images were then reconstructed by the BASEX.^38^ Tunable nanosecond laser pulses were generated from an ND:YAG laser system equipped with multiple harmonic generators (NT342, Ekspla) for the photodetachment and PHOFEX spectra. Femtosecond laser pulses, used for resonant two-photon photoelectron spectroscopy, were generated from the Ti:sapphire regenerative amplifier (Legend Elite-P, Coherent), which was seeded by the femtosecond oscillator (Vitara-T-HP, Coherent).

### Computational details

The ground state geometries of anions (D_0_) and neutrals (S_0_) were optimized to obtain the thermodynamic threshold energies for each dissociation reaction channel using the density functional theory (DFT) with the combination of the B3LYP functional and the 6-311++G(3df,3pd) basis set. The vertical excitation energies of D_0_ → D_*n*_ transitions were calculated using the time-dependent density functional theory at the same computation level as above. The first electronic excited-state (D_1_) of the nitromethane anion was optimized using the resolution-of-the-identity second-order approximate coupled-cluster singles and doubles (RICC2) method with the aug-cc-pVTZ basis set. In order to illustrate the C–N bond dissociation reaction of the nitromethane anion, potential energy curves of D_0_ and D_1_ states were obtained by scanning the C–N bond length while the other molecular geometries are kept frozen at the D_0_ equilibrium geometry. All DFT or RICC2 calculations were performed using the Gaussian 09 (ref. 39) or Turbomole 7.0.2 (ref. 40) program package.

## Results and discussion

**Fig. 1:**
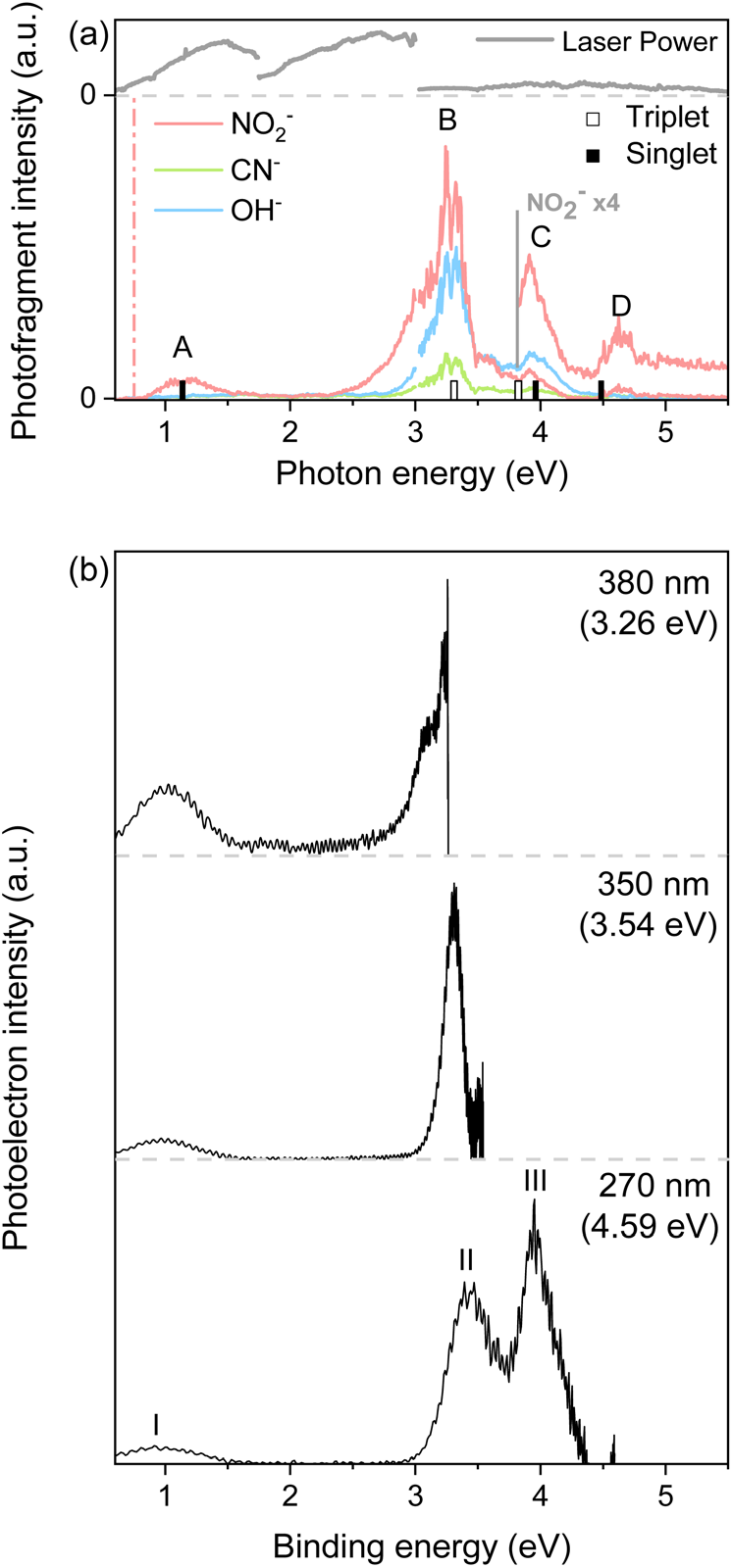
(a) PHOFEX spectra of the cryogenically-cooled nitromethane anion across the range of 0.6–5.5 eV. The spectra were acquired in four segments due to the laser configuration, with divisions at a photon energy of 1.75, 3.00 and 3.50 eV. Due to large fluctuations in laser power within our spectral window, laser power is represented in the upper panel. The theoretical vertical detachment energies to triplet (white column) and singlet (black column) states are depicted. The theoretical dissociation threshold for the NO_2_^−^ fragment is indicated with a vertical dashed line. (b) Photoelectron spectra of the nitromethane anion recorded at wavelengths of 380 nm, 350 nm, and 270 nm.

PHOFEX spectra of the cryogenically cooled nitromethane anion, taken for the anionic fragments of NO_2_^−^, OH^−^, and CN^−^ as a function of the excitation energy, are given in Fig. 1. The PHOFEX spectrum of NO_2_^−^ reflecting its fragmentation yield from the optical excitation of CH_3_NO_2_^−^ gives four distinct bands of A, B, C, or D centered on 1.04, 3.26, 3.95, or 4.68 eV, respectively. PHOFEX spectra of OH^−^ and CN^−^ exhibit similarly shaped bands of B, C, and D whereas the A band is observed only for NO_2_^−^, indicating that the thermodynamic appearance threshold for the OH^−^ (ref. 41) or CN^−^ fragment should be substantially higher than that for the NO_2_^−^ fragment. As the PHOFEX signal of the F-fragment (*S*_F_) as a function of the excitation energy (*E*) is supposed to be proportional to the absorption cross-section of the parent anion (*σ*_P_) times the relative yield of F (*P*_F_), giving the relation of *S*_F_(*E*) ∝ *σ*_P_(*E*)·*P*_F_(*E*), overall shapes of the individual PHOFEX bands should be governed by the absorption profiles of the parent anion unless the relative yields of the particular anionic fragments vary drastically within the same absorption band.

**Fig. 2:**
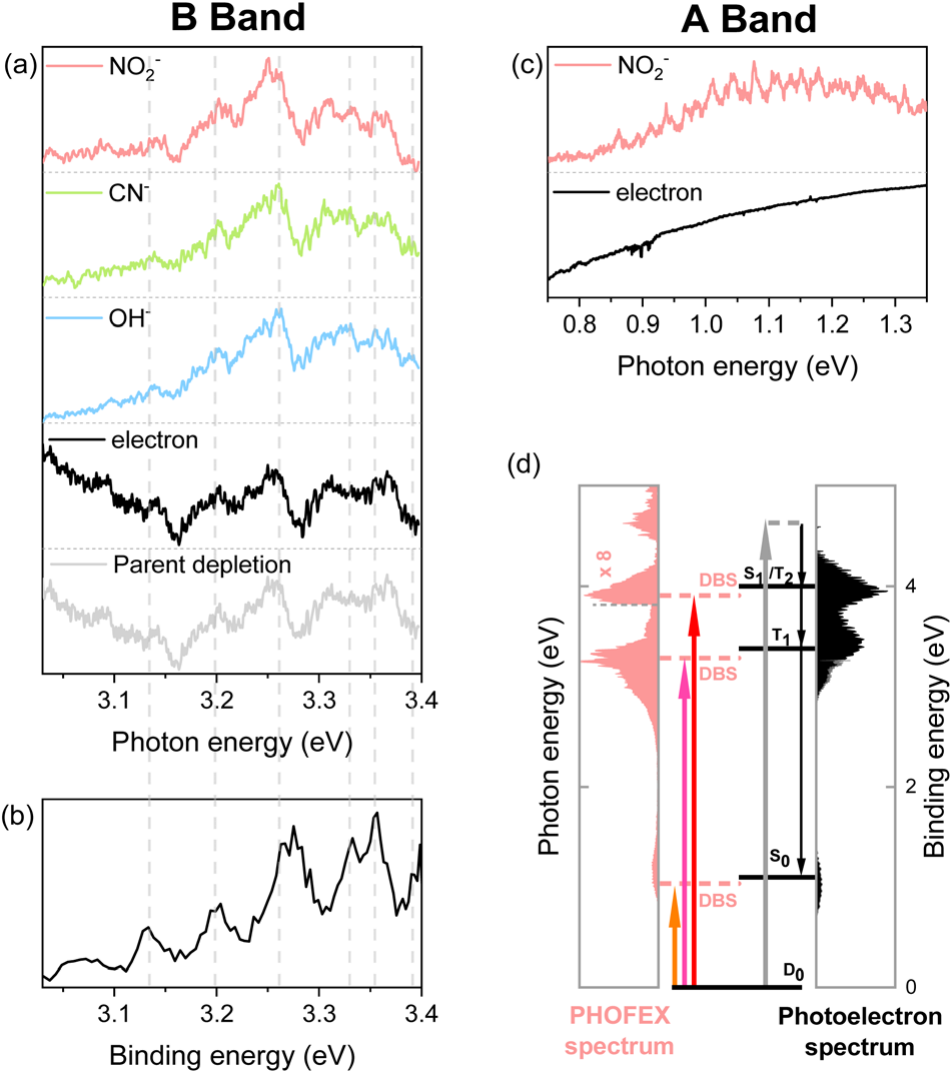
(a and c) PHOFEX and photodetachment spectra of the cryogenically cooled nitromethane anion in the B band (a) and A band (c) region, respectively, acquired while scanning the photon energy in 4 cm^−1^ steps. The photodepletion spectrum is also recorded for the B band. Signal intensities are normalized for clarity. (b) The partial 355 nm photoelectron spectrum of the nitromethane anion in the band II region (3.03–3.40 eV) by Sanov *et al.* (ref. ^34^). (d) Schematic energy-level diagram showing the anionic ground state, DBSs, and neutral states for nitromethane. The PHOFEX spectrum measured with NO_2_^−^ fragments and the photoelectron spectrum recorded at 270 nm are plotted. Below the dashed line at 3.26 eV, photoelectron spectrum data are replaced with the 380 nm photoelectron spectrum to accurately describe the lower neutral states (S_0_ and T_1_).

It should be noted that the electron affinity (EA) of CH_3_NO_2_^−^ is quite low, as it has recently been estimated to be ∼0.172 eV,^42^ and thus the photodetachment liberating the electron from the anion upon the optical excitation should be the most efficient process. And yet, as the photodetachment (or photoelectron) yield is the consequence from the optical excitation to the deionization continuum and also because it is cumulative with increasing the excitation energy, the Gaussian-shaped PHOFEX bands (Fig. 1) cannot be attributed to the dynamic behavior of the photodetachment process. The first impression might be then that the A-band (centered at 1.04 eV) could be due to the excitation to the first electronically excited-state (D_1_) of CH_3_NO_2_^−^. Despite that the possible contribution of the first-excited nitromethane anionic state to the A-band may not be completely excluded, there are several pieces of evidence against such a scenario. Namely, the quantum-mechanical calculations for the valence excited-states of the nitromethane anion do not reproduce the overall spectral structure of the PHOFEX spectrum (see ESI†). The PHOFEX bands could be then due to the optical transition to the dipole-bound states associated with the ground (A) and excited^43^ (B–D) states of the nitromethane neutral-core. It should be emphasized that the presence of DBS^30,32,36,42^ as well as its ultrafast relaxation^25,37^ into the anionic state of the nitromethane anion has already been reported in previous other experimental studies, suggesting that the optical transition to the DBS may end up with the efficient relaxation into the valence states of CH_3_NO_2_^−^, eventually leading to anionic fragmentation into the CH_3_ + NO_2_^−^ products, for instance. Namely, as the DBS associated with the ground neutral-core state survives only briefly with a lifetime of ∼400 fs,^25,37^ the relatively slow autodetachment process should be kinetically less favored while the ultrafast internal conversion of the DBS into the anionic valence state becomes facilitated. Accordingly, the spectral features of Feshbach resonances of the lowest DBS are not expected to stand out in the photodetachment spectroscopy (Fig. 2), although those are not anticipated to be identifiable either for the core-excited DBSs. The PHOFEX spectrum where the NO_2_^−^ yield represents the excitation profile to the DBS may then carry the vibronic structures of the meta-stable resonant states. Indeed, in the PHOFEX-A band (also in B-band; *vide infra*), many sharp resonant spectral features could be identified (Fig. 2) though the appropriate mode assignments are nontrivial as those are highly congested due to the much higher appearance energy of NO_2_^−^ (∼0.75 eV) compared to the EA threshold of 0.172 eV (*vide supra*).

Another strong evidence that the PHOFEX spectrum represents the D_0_ → DBS excitation is the experimental finding that the overall absorption profile is very similar to that of the photoelectron spectrum. It should be noted that the PHOFEX-A band is apparently truncated at the red-edge of the absorption profile due to the much higher NO_2_^−^ appearance threshold than the electron detachment threshold (*vide supra*). Actually, the photoelectron spectra of the cryogenically cooled nitromethane anion obtained at the excitation energy of 380, 350, or 270 nm (Fig. 1) give three distinct photoelectron bands centered at the binding energy of 0.97, 3.39, and 3.95 eV, which are in excellent agreement with the PHOFEX A, B, and C bands, respectively. In fact, those photoelectron bands of I (0.97), II (3.39) or III (3.95) correspond to the vertical detachment energies (VDEs) estimated for three lowest neutral states of S_0_, T_1_ and T_2_ of CH_3_NO_2_, respectively, according to the report by Sanov and co-workers,^34^ although the photoelectron band associated with S_1_ near the photoelectron band-II (Fig. 1) seems to be nearly absent in the present photoelectron spectrum. It should also be noted that all PHOFEX bands match very well with theoretically predicted vertical detachment energies (Table 1). As the DBS is (intrinsically) nearly identical to the neutral-core in terms of the molecular structure and energetics,^19^ the fact that the spectral pattern of the PHOFEX spectrum matches with that of the photoelectron spectrum strongly indicates that the former originates from the excitation to the DBS in the wide excitation energy range (0.5–5 eV). It should be then followed by the internal conversion to the low-lying valence states of the parent anion eventually leading to NO_2_^−^, OH^−^ or CN^−^ fragments. For the more accurate assignment of the PHOFEX-B band, for instance, a more thorough comparison between PHOFEX and photoelectron spectra has been made in terms of the detailed vibrational structure (Fig. 2). The highly resolved photoelectron band-II from ref. 34 where the Sanov group had assigned as the triplet state (T_1_) of nitromethane has been found to be in excellent agreement with the PHOFEX-B band, exhibiting the clear progressions and combinations of ∼240 and/or ∼540 cm^−1^ vibrational modes (see ESI†). Particularly, the 540 cm^−1^ mode assigned as the most Franck–Condon active NO_2_ bending matches quite well with the theoretical prediction of 547 cm^−1^.^34^ The PHOFEX-C band associated with the photoelectron-III band may be then attributed to S_1_ and/or T_2_ of nitromethane, whereas the PHOFEX-D band might reflect S_2_ (Table 1) though it is not unambiguous as the corresponding photoelectron spectrum is not available at the present time.

**Table 1:** Experimental and theoretical vertical transition energies for the D_0_ → neutral and D_0_ → DBS of nitromethane

Photoelectron band	Neutral state	Experimental D_0_ → neutral (eV)	Theoretical D_0_ → neutral (eV)	PHOFEX band	Experimental D_0_ → DBS (eV)
I	S_0_	0.97	1.14	A	1.04
II	T_1_	3.39	3.31	B	3.26
III	T_2_/S_1_	3.95/4.12^a^	3.82/3.96	C	3.95
	S_2_		4.49	D	4.68

a Ref. 34.

It should be noted that the determination of the D_0_-DBS transition oscillator strengths or the fragmentation yields is nontrivial at the present time. Nevertheless, experimental observation of the core-excited DBSs where the excess electron is attached to the excited-states of the neutral-core is quite novel. This also implies that the anionic fragmentation reactions upon the optical excitation, even in the complicated circumstances of the persistence of the detachment continuum, may take place mainly through the DBSs playing as the dynamic doorways. The fragmentation channel responsible for the PHOFEX-A band should then proceed as follows: 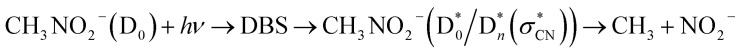. The bond dissociation would occur either statistically on 
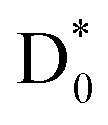
 (slow) or promptly on the optically dark 
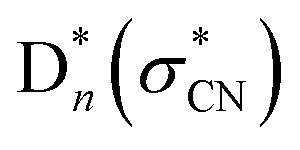
 state (fast). It has been found that many valence excited-states of the nitromethane anion are very crowded in the narrow excitation energy range, and it seems to be quite challenging to designate any particular excited-state responsible for the specific fragmentation channel.

And yet, the presence of 
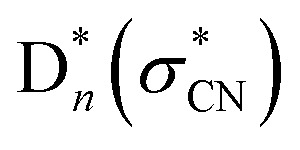
 which is repulsive with respect to the C–N bond elongation coordinate could be confirmed from our potential energy surface calculations (see ESI†). The NO_2_^−^fragmentation from 
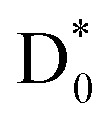
 (slow) would be less likely as the relatively faster vibrational autodetachment^44^ from 
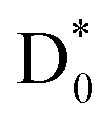
 should be kinetically dominant. In this sense, the NO_2_^−^ fragment from the PHOFEX-A band is most likely due to the ultrafast bond rupture taking place eventually on the 
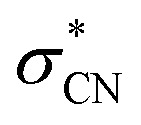
 state. All of NO_2_^−^, OH^−^, and CN^−^ fragments are produced from the PHOFEX-B, C, D bands, and it may result from the vibronic couplings among various electronic states of the anion. Incidentally, it is intriguing to note that the anionic fragmentation reaction originating from the core-excited DBS had not been reported to date. In this regard, the presence of the high-lying DBSs manifested by the PHOFEX-B, -C, or -D band strongly suggests that the DBS plays an important role as the doorway to the nonadiabatic transition even to the anionic excited-states, leading to the anionic fragments. Li *et al.* recently reported that the electron of the high kinetic energy range (4–8 eV) could be attached to the neutral peptide molecules (leading to the fragmentation) in the situation where the corresponding anionic valence states are hardly accessible.^45^ Accordingly, their electron-attachment spectrum was attributed to DEA possibly mediated by the core-excited DBSs. Our experimental finding supports such a scenario that the core-excited DBS may play an important dynamic role for the DEA occurring in the high collisional energy region. Besides, dynamics of the anionic fragmentation reactions associated with PHOFEX-B, C, D bands are certainly subject to the further investigation.

**Fig. 3:**
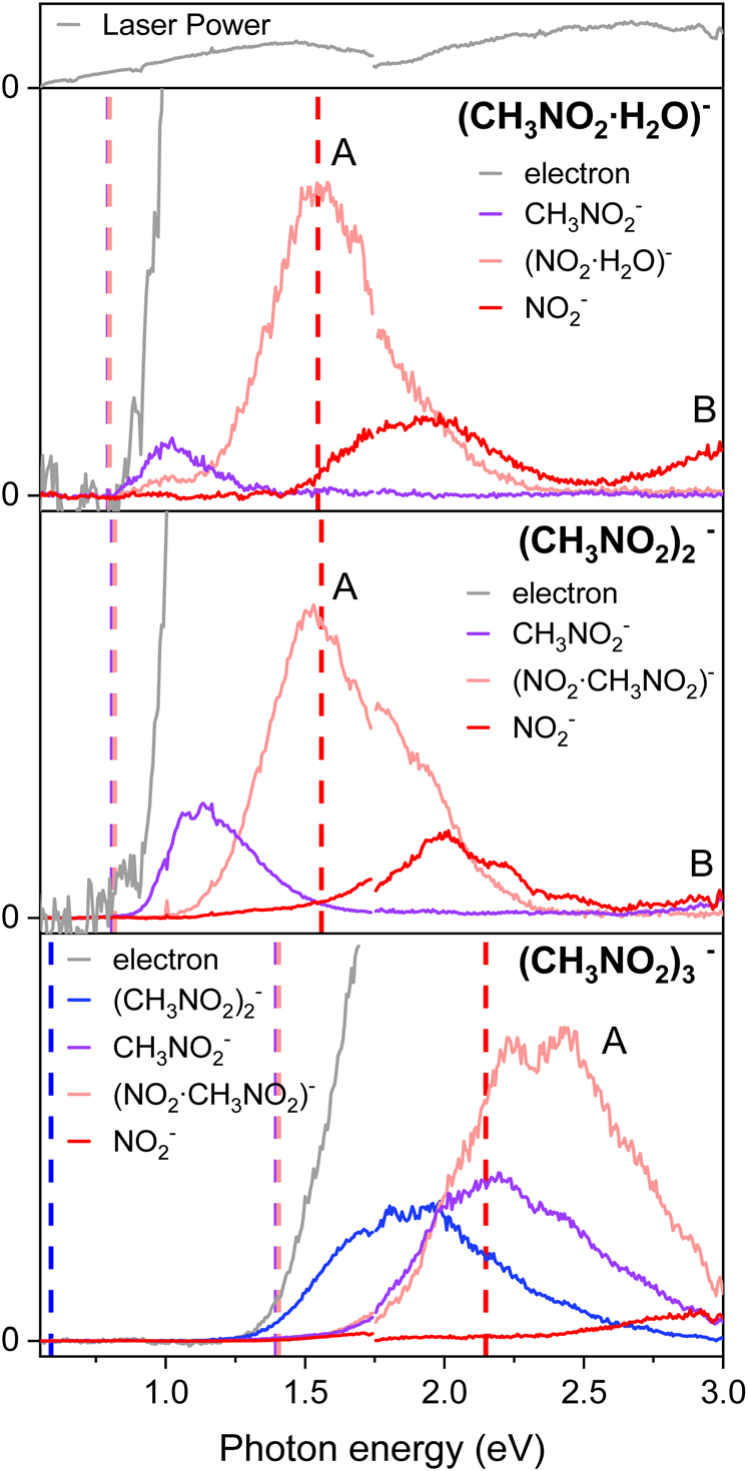
The PHOFEX and photodetachment spectra of cryogenically cooled (CH_3_ NO_2_·H_2_O)^−^, (CH_3_NO_2_)_2_^−^, and (CH_3_NO_2_)_3_^−^. Due to the laser configuration, the spectra were acquired in two segments with a division at photon energy of 1.75 eV and the laser power is represented at the top. The theoretical dissociation threshold values for each fragment are indicated with vertical dashed lines of the corresponding colors. Details of the calculations are described in the ESI.†

For the anionic clusters of (CH_3_NO_2_·H_2_O)^−^, (CH_3_NO_2_)_2_^−^, or (CH_3_NO_2_)_3_^−^ the PHOFEX spectrum of each species gives excitation profiles quite similar to those of the nitromethane anion in the 0.55–3.0 eV range (Fig. 3). Anionic fragments from the (CH_3_NO_2_)_2_^−^ dimer, for instance, consist of CH_3_NO_2_^−^, (CH_3_NO_2_)·NO_2_^−^, or NO_2_^−^ which result from the solvent (CH_3_NO_2_) evaporation, C–N bond rupture, or the combination of these, respectively. The PHOFEX excitation profiles of those anionic fragment species are different in terms of their appearance/disappearance energies. The CH_3_NO_2_^−^ PHOFEX band (due to the evaporation of the solvent) from the dimer anion starts to appear at ∼0.8 eV whereas its asymmetric-Gaussian-shaped excitation profile diminishes at ∼1.7 eV. The appearance energy of the CH_3_NO_2_^−^ fragment coincides with that of the photoelectron, suggesting that the corresponding PHOFEX signal may indeed originate from the excitation to the NBS of the anion dimer. It should be noted that the electron affinity of (CH_3_NO_2_)_2_^−^ is much higher than that of CH_3_NO_2_^−^ as the ground anionic state is much stabilized by the neutral nitromethane solvation.^35^ The blue-shifted (compared to the monomer) D_0_ → NBS transition of the dimer anion is thus likely to be responsible for the solvent–solute anion dissociation also because the otherwise direct absorption to the vibrationally hot 
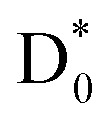
 of the anion dimer from the ground state is not expected to be significant. The CH_3_NO_2_^−^ fragmentation from (CH_3_NO_2_)_2_^−^ should result from the vibrational excitation of van der Waals modes in the charge–dipole interaction potential, most probably *via* the nonadiabatic transition from the NBS to 
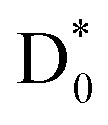
 of the dimer anion. It should be noted that the photoelectron spectra of the cluster anions reported in the previous other work^35^ are nearly identical to the PHOFEX spectra obtained in this work, strongly suggesting that the PHOFEX spectra should represent the D_0_ → NBS transitions of the cluster anions (Table 2). It is interesting to note that the vibrational spectral features observed in the PHOFEX-A band of the monomer anion (*vide supra*) could not be observed in the PHOFEX spectra of the cluster anions. This should be due to the spectral congestion caused by the high density of van der Waals modes of the latter. For the (CH_3_NO_2_)·NO_2_^−^ fragment channel, the Gaussian-shaped PHOFEX profile (1.1–2.2 eV) should then represent the D_0_ → NBS transition followed by the C–N bond rupture on the repulsive 
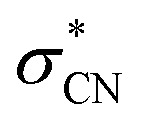
 state, as similarly found in the case of the monomer anion (*vide supra*). Therefore, the PHOFEX profile of the (CH_3_NO_2_)·NO_2_^−^ fragment may reflect the coupling strength of the NBS to the repulsive 
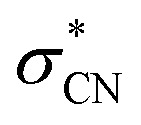
 state of the dimer anion as a function of the excitation energy. For the combination channel of the solvent evaporation and C–N bond rupture to give NO_2_^−^ from (CH_3_NO_2_)_2_^−^, as expected from the thermodynamic point of view, its PHOFEX yield shows the appearance threshold of ∼1.5 eV whereas it diminishes at ∼2.5 eV. The NO_2_^−^ fragment from the anion dimer could be the consequence from the sequential process of the C–N bond rupture followed by the solvent evaporation, although the detailed mechanism is subject to the further investigation. A similar systematic approach could be applied for the anionic cluster of (CH_3_NO_2_)_3_^−^ or (CH_3_NO_2_·H_2_O)^−^, and more detailed dynamics studies on those anion cluster systems will be forthcoming soon.

**Table 2:** Vertical transition energies for the D_0_ → S_0_ and D_0_ → NBS of the nitromethane clusters

Cluster	Theoretical D_0_ → S_0_ (eV)	Experimental^a^ D_0_ → S_0_ (eV)	Experimental D_0_ → NBS (eV)
(CH_3_NO_2_·H_2_O)^−^	2.0	1.7	1.6
(CH_3_NO_2_)_2_^−^	1.9	1.7	1.6
(CH_3_NO_2_)_3_^−^	2.4	2.2	2.2

a Ref. 35.

For the anion cluster of (CH_3_NO_2_)_2_^−^ or (CH_3_NO_2_)_3_^−^, for example, it could be arguable if its DBS could exist as the dipole moment of the nitromethane dimer (as the neutral-core) may be far below the conventional threshold value of 2.5 D (ref. ^18^ and 46) required for the presence of the DBS. It should be though emphasized that there have been a significant number of cases where other types of NBSs such as the CBS have been identified for the anionic cluster species.^10,24^ Therefore, it is highly plausible for the anionic clusters studied here to be present as the NBS. In order to validate such a scenario, we have performed resonant two-photon photoelectron spectroscopy using the femtosecond (fs) laser pulse. Because of the ultrashort lifetime of the NBS species, the photoelectron from the autodetachment of the NBS should be hardly detectable (*vide supra*). Using the fs laser pulses (Δ*t* ∼100 fs), we could clearly observe the anisotropic photoelectron band with the zero-binding energy from the two-photon excitation of the (CH_3_NO_2_)_2_^−^ or (CH_3_NO_2_)_3_^−^ at the respective photon energy of ∼0.83 eV or ∼1.55 eV (see ESI†). Here, the first photon excites the ground anion to the NBS whereas its loosely attached excess electron is detached by the second photon within the same fs laser pulse. This experimental finding strongly indicates that the NBS of the nitromethane dimer or trimer anion actually exists. From the fact that the dipole moments of the cluster species of nitromethane are quite low, the correlation effect is most probably responsible for the electron binding of the NBS species of these cluster anions.

## Conclusions

Anion PHOFEX spectroscopy has been employed to give the first experimental observation of the ground and core-excited dipole-bound states of the cryogenically-cooled nitromethane anion (CH_3_NO_2_^−^) which are associated with the S_0_, T_1_ or T_2_ state of the neutral-core. The excitation profiles reflected in the PHOFEX spectra represent the D_0_ → DBS transitions responsible for the anionic fragmentation reactions, indicating that the DBS may play the doorway role in the anionic fragmentation reaction leading to NO_2_^−^, OH^−^, or CN^−^. The overall spectral features of the PHOFEX bands are in excellent agreement with those of the photoelectron bands, strongly indicating that the transfer of the excess electron in the nonvalence orbital (of DBS) to the valence orbital (of anion) should be largely responsible for the anionic fragmentation channels. Photofragment action spectra of the anionic clusters of (CH_3_NO_2_)_2_^−^, (CH_3_NO_2_)_3_^−^, or (CH_3_NO_2_·H_2_O)^−^ give essential information regarding the complicated fragmentation reactions resulting from the electron transfer from the metastable NBS to the excited-states of the cluster anions.

## Data availability

The datasets generated and/or analyzed during the current study are available from the corresponding author on request.

## Author contributions

S. A. and D. K. conducted whole experiments. S. A. wrote the paper. J. K. performed computations. S. K. K. conceived the core idea, supervised the whole projects, and edited the manuscript.

## Conflicts of interest

There are no conflicts to declare.

## Supplementary Material

SC-014-D3SC04342H-s001
