# Supplementary material for: Excited-state chemistry of the nitromethane anion mediated by the dipole-bound states revealed by photofragment action spectroscopy
Source: Chem Sci. 2023 Oct 16;14(43):12231–7. doi: 10.1039/d3sc04342h (PMC10631229; doi:10.1039/d3sc04342h)
Supplement: SC-014-D3SC04342H-s001 [file SC-014-D3SC04342H-s001.pdf]

Electronic Supplementary Material (ESI) for Chemical Science.
This journal is © The Royal Society of Chemistry 2023

Supplementary Information

Excited-state chemistry of the nitromethane anion mediated by the dipole-bound states revealed by the photofragment action spectroscopy

Sejun An, Dabin Kim, Junggil Kim, and Sang Kyu Kim*

Department of Chemistry, KAIST, Daejeon (34141), Republic of Korea

*Corresponding author: sangkyukim@kaist.ac.kr

Table of Contents

1. Theoretical Calculations of the Valence Excited States of the Nitromethane Anion

2. Vibrational Structure Analysis of the PHOFEX-B band

3. Femtosecond Resonant Two-photon Photoelectron Spectrum (R2PES) of the $(CH_3NO_2)_2^-$ and $(CH_3NO_2)_3^-$

4. The Time-of-flight Photofragment Mass Spectra of the Nitromethane and Its Cluster Anions

5. DFT Calculations for the Thermodynamic Dissociation Threshold Values

1. Theoretical Calculations of the Valence Excited States of the Nitromethane Anion

The vertical excitation energies of the valence states ($D_0 \rightarrow D_n$ transition) were calculated at the TD-DFT/B3LYP/6-311++3df level (Fig. S1). The calculated values significantly diverge from the overall spectral structure of the PHOFEX spectrum. However, the vertical excitation energy of the $D_0 \rightarrow D_1$ transition (1.02 eV) is close to the maximum of the PHOFEX-A band (1.1 eV). Given that the PHOFEX spectrum closely follows the Franck-Condon distribution of the nonvalence-bound state (NBS) and shows significant discrepancy with other $D_0 \rightarrow D_n$ transitions, it is highly probable that this correspondence is coincidental. Nonetheless, we cannot completely rule out the possibility that the PHOFEX-A band originated from $D_0 \rightarrow D_1$ transition, prompting us to attempt additional calculations

First, one-dimensional potential energy curves of the nitromethane anion for the D_0 and D_1 states along the C-N bond extension coordinate were calculated at the RI-CC2/aug-cc-pVTZ level (Fig. S2). Both D_0 and D_1 are found to be bound states in the Franck-Condon region, as they possess π^* and 3*s**(carbon) orbital configurations at the equilibrium geometries, respectively. As the C-N bond length is elongated, the D_0 state is the only state adiabatically correlating to the $CH_3\cdot$ + NO_2^-(X) channel. To interrogate which types of electronic states are contributing to the dissociation process, we calculated the SOMO orbital of D_0 state at the particular C-N bond length, shown in the inset of Fig. S2. Notably, the orbital configuration of the SOMO undergoes a gradual change from π^* to σ_{CN}^* types with the increase of C-N bond length, particularly at the R_{CN} = 2.00 Å, indicating that the D_0 state bound in the Franck-Condon region should be crossed by a repulsive σ_{CN}^* state to correlate to the ground state product channel in the asymptotic region (dotted line). This strongly suggests that the C-N bond dissociation reaction from the NBS should be effectively facilitated by σ_{CN}^* state.

Subsequently, the vertical and adiabatic excitation energies of $CH_3NO_2^-$ and $(CH_3NO_2\cdot H_2O)^-$ for the $D_0 \rightarrow D_1$ transition were calculated from the optimized geometry of the D_0 and D_1 states (Table S1). For the $(CH_3NO_2\cdot H_2O)^-$, the adiabatic excitation energy is much larger than experimentally observed appearance energy, which cannot result in the broad PHOFEX-A band. For the nitromethane anion, the PHOFEX-A band locates near the dissociation threshold, leading to a truncated red-edge. This can be confirmed through its asymmetrical Gaussian shape (Fig. S3), and it is apparent that the absorption band is indeed broader, which is again inconsistent with the small difference between adiabatic and vertical energies for the $D_0 \rightarrow D_1$ transition. Although theoretical calculations can provide shifted values, the difference between the vertical and adiabatic excitation energy values is generally accurately

predicted. Given the significant discrepancy with the experimental values, we can conclude that the absorption band is not due to the $D_0 \rightarrow D_1$ transition. On the other hand, difference between vertical and adiabatic detachment energies of the $D_0 \rightarrow S_0$ transition is consistent with experimental values which demonstrates the validity of our calculation method.

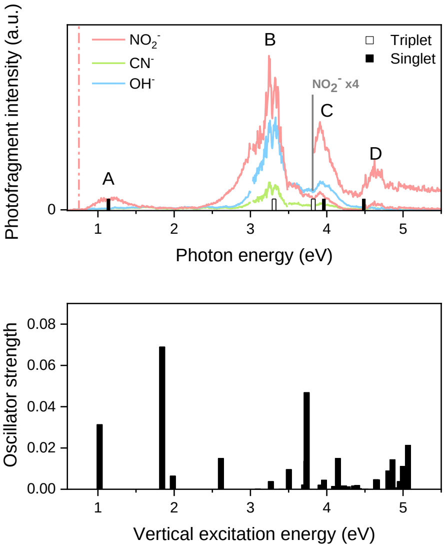

Fig. S1 TD-DFT calculation results for the vertical energies of the $D_0 \rightarrow D_n$ transitions. The PHOFEX spectra are plotted for the comparison.

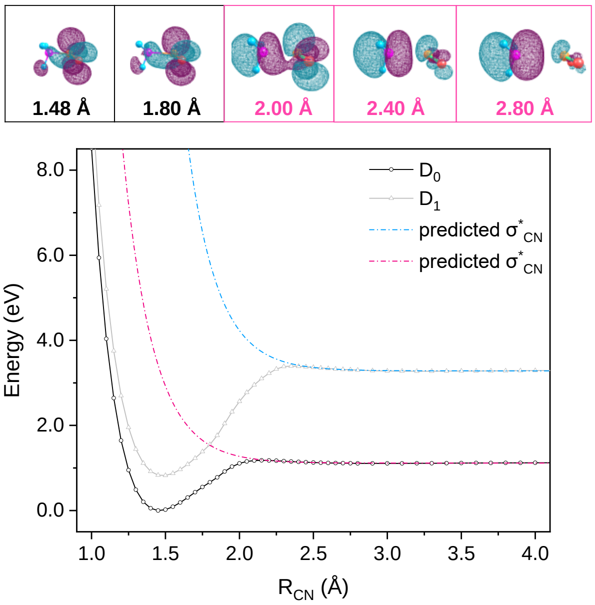

Fig. S2 One-dimensional potential energy surfaces for the D_0 and D_1 states, which are obtained by rigid scanning along the C-N bond extension coordinate of the nitromethane anion. Predicted σ_{CN}^* states are represented in the dashed line. Calculated SOMO orbital configurations of the D_0 states are described on the top panel.

Table S1 Adiabatic and vertical excitation energies, along with their differences, were calculated at the RI-CC2/aug-cc-pVTZ level. Values calculated with the aug-cc-pVDZ basis set are provided in parentheses**.** Employing the same method, the difference between adiabatic (ADE) and vertical (VDE) detachment energies were calculated, which is consistent with the PHOFEX and photoelectron spectrum.

Clusters	Adiabatic D_0-D_1 (eV)	Vertical D_0-D_1 (eV)	Difference (eV)	Theoretical VDE-ADE (eV)	Experimental VDE-ADE (eV)[a]
$CH_3NO_2^-$	0.63(0.84)	0.83(0.94)	0.20(0.10)	0.61(0.63)	0.7[a]
$(CH_3NO_2{\cdot}H_2O)^-$	1.14(1.32)	1.50(1.56)	0.36(0.24)	0.81(0.88)	0.9[b]

[a] Reference [1], [b] Reference [2]

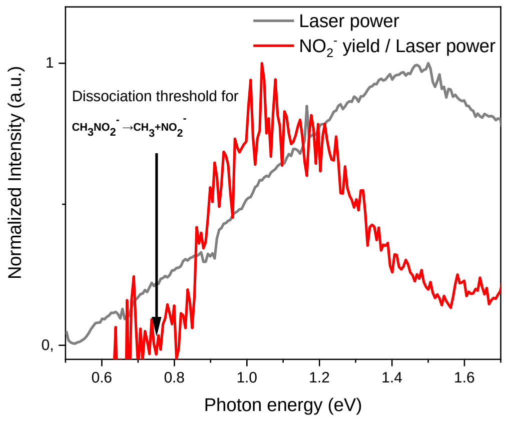

Fig. S3 The laser power-corrected spectrum for the PHOFEX-A band. The red-edge of the A band was initially underestimated due to the laser power; therefore, a correction was applied by dividing by the laser power. Despite this, truncation is still observed when compared to the blue-edge. This suggests that the appearance of an asymmetric Gaussian band is due to the dissociation threshold energy and implies that an absorption band begins at lower energy.

2. Vibrational Structure Analysis of the PHOFEX-B band

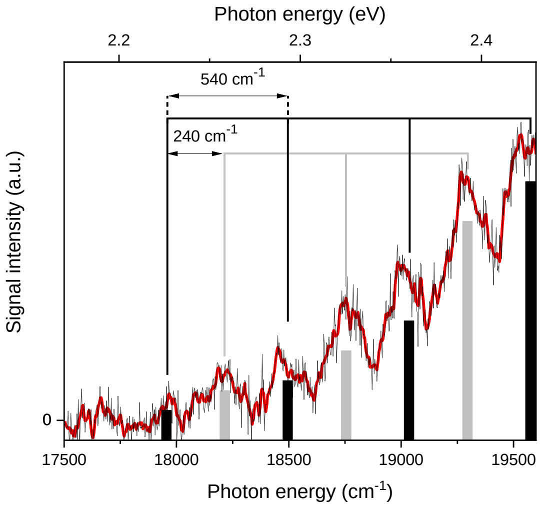

Fig. S4 The PHOFEX spectrum with the NO_2^- fragment of the nitromethane anion at the photon energy of 2.17 ~ 2.43 eV. Stick spectra for two vibrational progressions, with a common origin at 2.23 eV and frequencies 240 cm^{-1} and 540 cm^{-1}, are indicated. The red line represents a 10-point averaged smoothing data.

3. Femtosecond Resonant Two-photon Photoelectron Spectrum (R2PES) of the $(CH_3NO_2)_2^-$ and $(CH_3NO_2)_3^-$

Using femtosecond laser pulses, the two-photon detachment signal via the NBS was obtained from $(CH_3NO_2)_2^-$ and $(CH_3NO_2)_3^-$ (Fig. S4). To reduce the nonresonant photoelectron signal, the photon energy was tuned to near the detachment threshold of the dimer and trimer anion, 0.83 eV and 1.55 eV, respectively. The dimer anion exhibits a sharp NBS peak, which is clearly distinguished from the nonresonant detachment band by the binding energy. In contrast, the trimer anion yields a poor quality of R2PES due to the small output from our ion source. Nonetheless, resonant two-photon signal via the NBS are easily distinguishable from nonresonant multiphoton signals. Based on the binding energy of the trimer anion, we would expect a nonresonant three-photon photoelectron band centered at a binding energy of -0.9 eV. However, only noise signal exists below -0.5 eV, enabling us to rule out the processes involving more than three photons. Based on a recently reported photoelectron spectrum[2], a simulated nonresonant two-photon photoelectron band with a maximum of 0.65 eV is plotted in Fig. S4. This simulated band significantly deviates from the observed photoelectron signal near 0 eV. Therefore, it is evident that our photoelectron spectra, obtained using focused femtosecond laser pulses, cannot be attributed to a nonresonant multiphoton process but rather corresponds to a resonant two-photon process via the NBS.

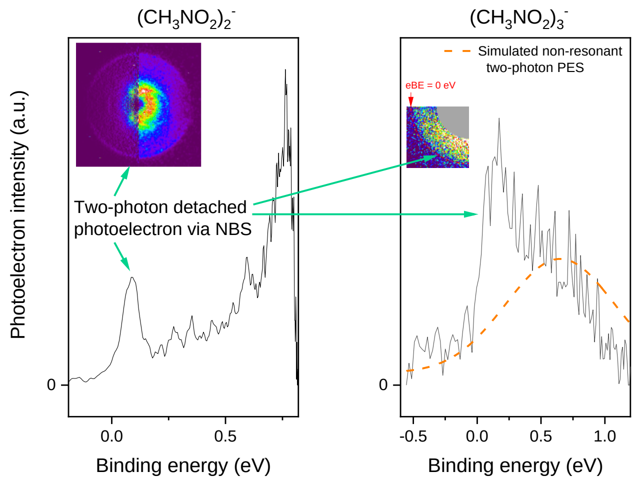

Fig. S5 Femtosecond resonant two-photon photoelectron spectra of the $(CH_3NO_2)_2$- and $(CH_3NO_2)_3^-$. For the dimer anion, the reconstructed (left) and raw (right) images are displayed in the inset. The green arrows indicate two-photon detached photoelectron signal via the NBS in the photoelectron images and spectra. The central part of both images, which corresponds to higher binding energy, is distorted due to the saturation effect on the CMOS camera. However, the outer part of the image remained unaffected, allowing for clear observation of the NBS peaks.

4. The Time-of-flight Photofragment Mass Spectra of the Nitromethane and Its Cluster Anions.

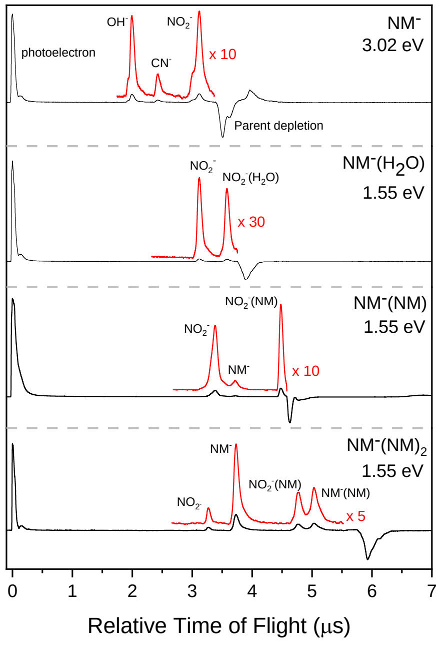

Fig. S6 The time-of-flight photofragment mass spectra of nitromethane and its cluster anions are recorded by the difference between laser-on/off signals. The relative time-of-flight is depicted based on the arrival time of the photoelectron.

5. DFT Calculations for the Thermodynamic Dissociation Threshold Values

The appearance energies of each fragment are calculated from the thermodynamic dissociation threshold values based on following equations at the B3LYP/6-311++3df3pd level

· $CH_3NO_2^- \rightarrow CH_3 + \underline{NO_2^-}$

· $(CH_3NO_2{\cdot}H_2O)^- \rightarrow H_2O + \underline{CH_3NO_2^-}$

$\rightarrow CH_3 + \underline{(NO_2{\cdot}H_2O)^-}$

$\rightarrow CH_3 + H_2O + \underline{NO_2^-}$

· $(CH_3NO_2)_2^- \rightarrow CH_3NO_2 + \underline{CH_3NO_2^-}$

$\rightarrow CH_3 + \underline{(NO_2{\cdot}CH_3NO_2)^-}$

$\rightarrow CH_3 + H_2O + \underline{NO_2^-}$

· $(CH_3NO_2)_3^- \rightarrow CH_3NO_2 + \underline{(CH_3NO_2)_2^-}$

$\rightarrow 2{\cdot}CH_3NO_2 + \underline{(CH_3NO_2)^-}$

$\rightarrow CH_3 + CH_3NO_2 + \underline{(NO_2{\cdot}CH_3NO_2)^-}$

$\rightarrow CH_3 + 2{\cdot}CH_3NO_2 + \underline{NO_2^-}$

References

1. C. L. Adams, H. Schneider, K. M. Ervin and J. M. Weber, *The Journal of Chemical Physics*, 2009, **130**, 074307.
2. C. J. M. Pruitt, R. M. Albury and D. J. Goebbert, *Chemical Physics Letters*, 2016, **659**, 142-147.
